# Bilateral retinal toxicity as a result of poisoning with pure iodine

**DOI:** 10.22336/rjo.2021.14

**Published:** 2021

**Authors:** Esra Bahadir Camgoz, Yasin Sakir Goker

**Affiliations:** * Ophthalmology Department, University of Health Sciences Ulucanlar Eye Training and Research Hospital, Ankara, Turkey

**Keywords:** iodine toxicity, toxic retinopathy, optical coherence tomography, fundus autofluorescence

## Abstract

Iodine is an essential mineral that is necessary for the synthesis of thyroid hormones, which can cause many diseases in the body. The application of adding potassium iodate to table salts started in Turkey in 1998. High doses of iodate cause retinal toxicity, leading to significant vision loss. A 42-year-old paranoid schizophrenic patient who attempted suicide with pure iodine was admitted with bilateral vision loss. Widespread retinal pigment epithelium (RPE), ellipsoid zone (EZ) and interdigitation zone (IZ) damage were present in the optical coherence tomography (OCT) assessment. Fundus autofluorescence (FAF) findings, which included hypoautofluorescence areas that supported this condition, were also found.

In conclusion, iodate in high doses is toxic on RPE, EZ and IZ. This situation could be irreversible depending on the dose.

## Introduction

Iodine is an essential mineral that is extremely important for thyroid hormone synthesis, cognitive functions, growth, and development. In iodine deficiency, hypothyroidism, cretinism, physical and mental retardation, and abortion may occur [**1**,**2**]. It is one of the worldwide preventable causes of mental and physical developmental retardation [**3**].

Iodine table salt has been used for years to provide iodine support. Iodine addition to table salt officially started in Turkey in 1998 [**4**,**5**]. Potassium iodate is most used for this purpose. The ingested iodate is metabolized into iodide and taken up by the thyroid glands. High dose of iodate is toxic to retinal pigment epithelium (RPE) and photoreceptors [**6**].

In this case report, we presented a case that developed bilateral retinal toxicity as a result of poisoning with pure iodine, with clinical findings and imaging.

## Case report

A 42-year-old paranoid schizophrenic who worked in a salt factory was admitted to our clinic with bilateral vision impairment. Approximately 20-25 days before, the patient had attempted suicide with 2 cups of pure iodine. Afterwards, he had a second suicidal attempt by jumping high after his rescue, but he was later treated. For this reason, the physical condition of the patient was a challenge in the examination.

**Fig. 1:**
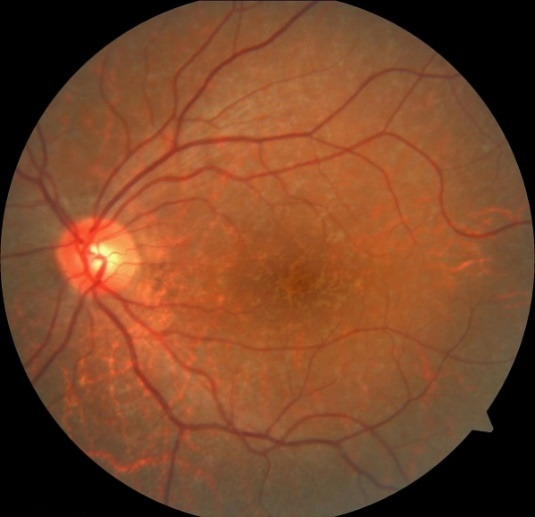
The patient’s left eye colored fundus photograph shows hypopigmented areas in the macula

**Fig. 2:**
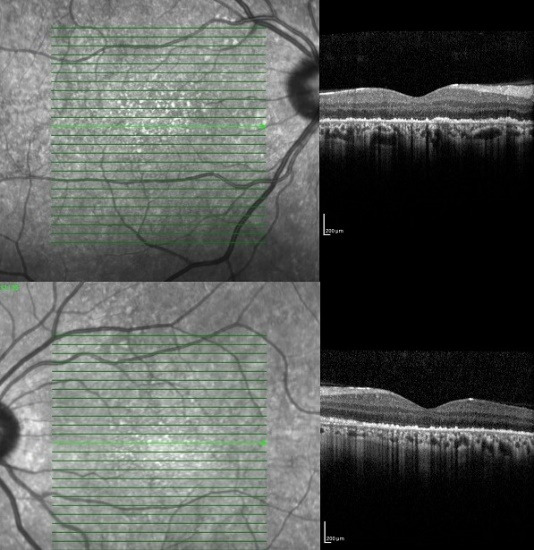
Outer retinal atrophy including loss of RPE and photoreceptor is observed in both eye OCTs of the patient

**Fig. 3:**
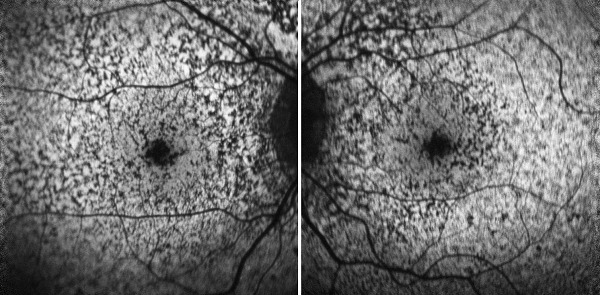
Hypoautofluorescent areas consistent with loss of RPE and photoreceptors are observed in both eyes FAF of the patient

In the ophthalmologic examination, the best corrected visual acuity in both eyes was at the level of finger counting from 2 meters. No pathology was detected during anterior segment examination with a biomicroscope. Intraocular pressures measured by pneumotonometry were at normal levels. During the fundus examination of the patient, significant hypopigmented areas were observed in the macula area in both eyes (**Fig. 1**). Afterwards, optical coherence tomography (OCT) and fundus autofluorescence (FAF) imaging were applied to the patient. Macular OCT imaging showed outer retinal atrophy involving widespread RPE and photoreceptor cell loss in both eyes (**Fig. 2**). In FAF imaging, widespread hypoautofluorescent areas were observed in both eyes, consistent with RPE and photoreceptor loss in the outer retina (**Fig. 3**). Retinal nerve fiber layer analysis (RNFL) was performed to detect possible optic disc damage, but no pathology was detected in this scan. Due to insufficient patient compliance, the images of optic coherence tomography angiography obtained were not optimal. No ophthalmologic disease was found in the patient’s and his family’s medical history. The patient’s prior military service duty and driver’s license were supportive of this situation.

## Discussion

The toxic effects of potassium or sodium iodate on the retina have been shown in many studies. Sodium iodate has been used to demonstrate RPE damage in many studies involving monkeys, pigs, rabbits, and sheep. It is experimentally capable of forming retinitis pigmentosa or age-related macular degeneration in animals. Also, in these animal studies, sodium iodate at doses of 20 to 80 mg/ kg was used to create retinal toxicity [**7**]. In an animal study, Moriguchi et al. have showed that retinal toxicity, due to sodium iodate, is reversible at low doses and irreversible at high doses [**8**]. In the case series of 5 patients, Singalavanija et al. have found that oral potassium iodate doses of 187 mg/ kg or more, in humans, caused retinal damage [**9**]. In our case, 2 cups of pure iodine intake were determined, but the dose of iodine could not be calculated clearly. The extensive photoreceptor loss indicated that the ingested dose might have been higher and that the damage was most likely irreversible. 

In the 5-case series of Singalavanija et al., patients were treated with prednisolone, vitamins B1, B6 and B12, and some patients had visual gain associated with the absorbed iodate dose [**9**]. However, in our case, intense photoreceptor loss in imaging and 1-month delayed admission indicated that the patient would not benefit from treatment. 

In another study on rats, RPE damage caused by sodium iodate and chorioretinal atrophy were evaluated by comparing FAF imaging with electroretinogram (ERG) findings and it was shown that FAF could be useful in these cases as a non-invasive test [**10**]. The FAF images in our case were demonstrative for the resulting RPE damage and supported that situation.

In the case report of Venu Gopal Reddy et al., initially the patient with bilateral visual acuity loss was researched for choroideremia, retinitis pigmentosa and posterior uveitis and then medical history was investigated deeply and realized that he had retinal toxicity related with suicidal attempt by iodate 10 years before [**6**]. From this point of view, it was seen that medical and family history were extremely important in diagnosing patients. In our case, asking about a driver’s license owning and military service history was useful to confirm that the cause of vision loss was related to iodine.

## Conclusion

Iodate-related toxic retinopathy occurs by affecting RPE, ellipsoid zones and interdigitation zones. In these cases, FAF may show damage noninvasively. Toxicity is dose dependent. Detailed questioning of the patients’ background and family history prevents the confusion of diagnosis in these cases.

**Conflict of Interest**

Authors state no conflict of interest.

**Informed Consent and Human and Animal Rights statements**

Informed consent has been obtained from all individuals included in this study.

**Authorization for the use of human subjects**

Ethical approval: The research related to human use complies with all the relevant national regulations, institutional policies, is in accordance with the tenets of the Helsinki Declaration, and has been approved by the institutional review board/ committee of the University of Health Sciences Ulucanlar Eye Training and Research Hospital, Ankara, Turkey.

**Acknowledgements**

None.

**Sources of Funding**

None.

**Disclosures**

None.
